# diel_models: a python package for systematic integration of day–night cycles into plant genome-scale metabolic models

**DOI:** 10.1093/bioadv/vbaf087

**Published:** 2025-04-16

**Authors:** Luciana Martins, João Capela, Emanuel Cunha, Marta Sampaio, Oscar Dias

**Affiliations:** Centre of Biological Engineering, University of Minho, Braga 4710-057, Portugal; Centre of Biological Engineering, University of Minho, Braga 4710-057, Portugal; Centre of Biological Engineering, University of Minho, Braga 4710-057, Portugal; Centre of Biological Engineering, University of Minho, Braga 4710-057, Portugal; Centre of Biological Engineering, University of Minho, Braga 4710-057, Portugal; LABBELS—Associate Laboratory, Braga, 4710-057, Portugal

## Abstract

**Summary:**

In recent years, genome-scale metabolic models have become indispensable tools for studying complex metabolic processes occurring within living organisms. Understanding plants’ metabolic behaviour under diel cycles (24-h day–night cycles) is essential to explain their adaptive strategies to different light conditions. However, integrating these cycles in plant GEMs is complex, laborious, time-consuming, and not systematized. Here, we present *diel_models*, a novel python package that enables the systematization and accurate construction of diel models based on non-diel plant GEMs, tailored for generic and multi-tissue models. *diel_models* is a lightweight, modular package with minimal dependencies and broad Python compatibility (v3.8+), making it easy to use, integrate into reconstruction pipelines, and extend with community-driven enhancements. It is also supported on all operating systems, including Windows, MacOS, and Linux, ensuring cross-platform compatibility for a wide range of users.

**Availability and implementation:**

The code is freely available at https://github.com/BioSystemsUM/diel_models.git and can be installed using the command pip install diel_models.

## 1 Introduction

Archaeal, bacterial, and eukaryotic metabolism can be computationally simulated using genome-scale metabolic models (GEMs). These models comprehensively represent all known metabolic reactions catalyzed by enzymes encoded in the genome and compile all known metabolic processes in the organism under study, including related enzymes and transporters (Thiele and Palsson 2010). GEMs integrate genomic data with biochemical knowledge to predict the behaviour of cellular metabolism under different conditions (Passi *et al.* 2022). As mathematical models, GEMs enable the simulation of an organism’s metabolism as a vast network of interrelated biological reactions. They allow for predicting metabolic flux values for a wide range of metabolic reactions using optimization approaches such as flux balance analysis (FBA) (Orth *et al.* 2010). By defining an objective function to minimise or maximize one or more reactions, GEMs can align closely with research questions, such as maximizing cellular growth or achieving other specific objectives (Rocha *et al.* 2008), like optimization of metabolite production, improving nutrient use efficiency, among others.

Although initially these models were reconstructed for bacteria (e.g. Edwards and Palsson 2000) and yeast (e.g. Förster *et al.* 2003), further research allowed recreating GEMs for various multicellular organisms, including humans (Duarte *et al.* 2007) and plants (de Oliveira Dal’Molin *et al.* 2010).

In the context of plant physiology, plant genome-scale metabolic models (pGEMs) facilitate the investigation of how a plant reacts to environmental changes, including nutrient availability (Postma *et al.* 2014), light intensity and temperature (Fu *et al.* 2019), and response to drought stress (Siriwach *et al.* 2020, Yuan *et al.* 2016). Although numerous pGEMs have been reconstructed and validated, there are still challenges and limitations (Sampaio *et al.* 2022).

As photoautotrophic organisms, plants depend on light to grow, performing photosynthesis to convert light energy into chemical energy stored in glucose molecules (Stirbet *et al.* 2019).

This process drives plants’ metabolism, enabling the production of essential compounds and supporting growth, reproduction, and cellular respiration. Light is not always constant, showing variations during a day-and-night cycle (diel). While photosynthesis occurs during the day, when light is available, the absence of light at night drastically alters plant cell metabolism. Due to this intricacy, pGEMs must incorporate diel cycles contrary to GEMs of other non-phototrophic organisms, though some mammals may also exhibit distinct metabolic shifts between feeding and fasting states.

Dynamic Flux Balance Analysis (dFBA) could consider these dynamic environmental conditions and their metabolic responses, as it enables the simulation of dynamic biological systems combining FBA, with ordinary differential equations (ODEs) to simulate changes in metabolite concentrations over time (Grafahrend-Belau *et al.* 2013). However, dFBA requires estimating several kinetic parameters; thus, integrating dFBA analyses in pGEMs becomes challenging (Gomez *et al.* 2014).

Therefore, several approaches have been used over the years to construct diel pGEMs. After the initial reconstruction of the *Arabidopsis thaliana* metabolic model in 2009 (Poolman *et al.* 2009), numerous revisions and implementations followed and are still published nowadays (Maurice Cheung *et al.* 2014, Shaw and Cheung 2018, Siriwach *et al.* 2020). The first model incorporating the simulation of day and night cycles in leaf metabolism was put forward for *A. thaliana* in 2014 (Maurice Cheung *et al.* 2014). Based on a prior model from 2013 (Cheung *et al.* 2013), the authors of this study recreated the diel model by developing two modules (representing the day and night phases). Transporters were manually inserted to enable the transfer of metabolites between the two modules. The model was simulated as a single optimization problem with precise constraints that enabled photon influx during the day and set it to zero at night. This model included a common storage pool of metabolites created during the day to be used at night and vice versa by considering the interactions between the two phases.

In 2018, a multi-tissue diel GEM of *A. thaliana* was constructed (Shaw and Cheung 2018) with the creation of a storage pool to allow the transition of metabolites between the different phases, day and night, and also a common pool for the transition of certain metabolites between the leaf and the root.

In addition to *A. thaliana* pGEMs, other organisms such as *Quercus suber* (Cunha *et al.* 2023) and *Vitis vinifera* (Sampaio *et al.* 2024) have also been the target of studies and creation of their multi-tissue diel models. In these two cases, a storage pool was also established between the different phases and tissues, allowing for the transition of the desired metabolites.

Despite successfully applying all of these steps to individual models, they have not been carried out systematically. To the best of our knowledge, there has been no approach capable of doing so in a single generic framework. Therefore, we hereby propose *diel_models*, a python package that consolidates all the necessary aspects for creating a diel model into a single-step process. The *diel_models* package supports both generic and multi-tissue models, offering flexibility for incorporating a range of additional relevant considerations, such as integrating other steps into the pipeline. It is accessible to the entire community via pip install diel_models, has only one associated dependency, and is compatible with all Python versions above 3.8. The *diel_models* project incorporates continuous integration and delivery (CI/CD) best practices to guarantee compatibility with Python versions above 3.8 and across all operating systems. Code quality is consistently verified using unit tests, and test coverage is assessed prior to each repository merge. Releases are automatically published to the Python Package Index (PyPI).

All Python code, documentation, validation, and simulatable plant diel models are available on GitHub at https://github.com/BioSystemsUM/diel_models.git.

## 2 Implementation

**Figure 1.**
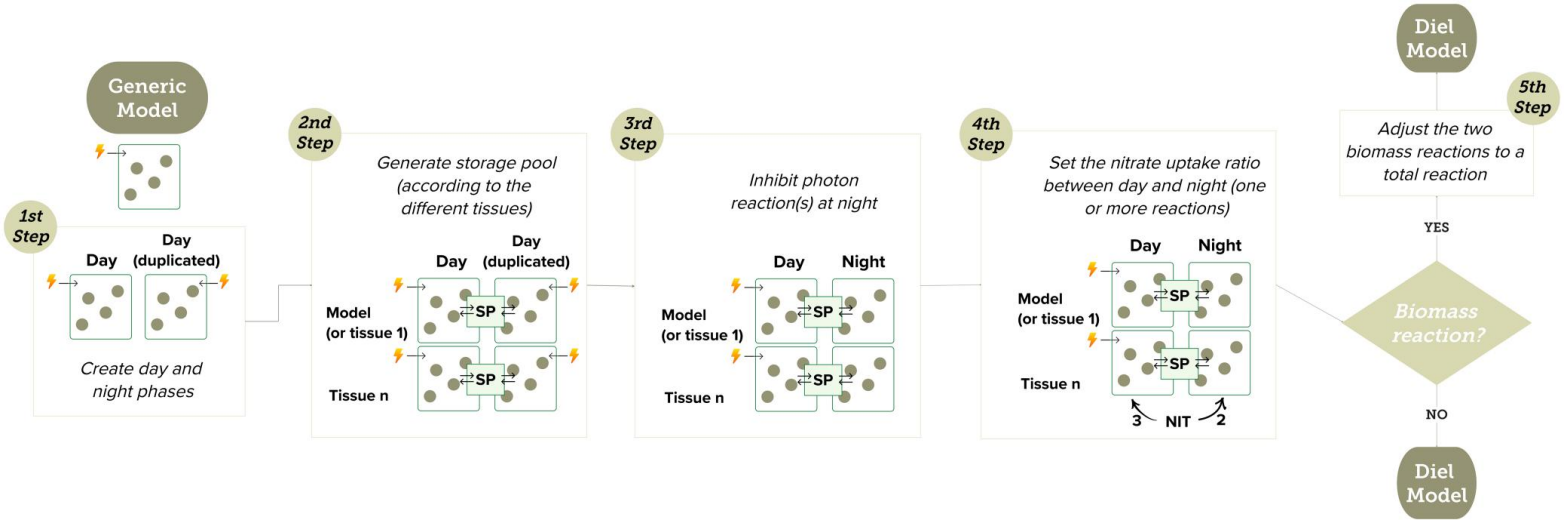
Diel models creator pipeline illustration. First step represents the creation of day and night phases by duplicating all compartments, reactions, and respective metabolites. The second step involves generating the storage pool, which will be tailored according to the specific tissues in the case of a multi-tissue model. This storage pool can contain, e.g. amino acids, carboxylic acids, nitrate, starch, and sugars. The third step involves inhibiting the reaction(s) of photon uptake (represented by the lightning) during the ‘day (duplicated)’ phase, effectively converting this compartment to the night phase. The fourth step is optional and involves setting up the nitrate uptake ratio between day and night (one or more reactions) according to a user-defined value, with a default ratio of 3:2. Fifth step is optional and represents the creation of another reaction that merges the day and night biomass reactions. SP, Storage Pool; NIT, Nitrate (for a more detailed schema see Supplementary Fig. S1).

*diel_models* consists of a pipeline that executes five steps in a predetermined sequence. When the original model does not contain a biomass reaction only four steps are required, as seen in Fig. 1.

The first stage (Fig. 1, first step) of the pipeline involves duplicating all compartments, reactions, and corresponding metabolites, using the non-diel model as input, producing ‘Day’ and ‘Night’ phases. The suffix in their names is the only element that differentiates the compartments, reactions, and metabolites of day and night (e.g. ‘Glucose_c’ to ‘Glucose_c_Day’ and ‘Glucose_c_Night’). In this step, it is not yet relevant whether the model is a multi-tissue model.

The second step is the storage pool generation step (Fig. 1, second step), which establishes a new compartment called ‘storage pool’ where metabolites can move from the day to the night phase and vice versa. Then, exchange reactions between day and night are also created. If the metabolic model includes multiple plant tissues, the user will have to provide their names. This allows *diel_models* to create a storage pool of metabolites for each tissue during this step.

The third step (Fig. 1, third step) involves blocking the photon uptake flux during the night. This step consists of setting the Upper Bound (UB) and Lower Bound (LB) of the photon uptake reaction(s) to zero. In this step, the flux of multiple reactions can be blocked because, in a multi-tissue model, multiple photon uptake reactions might be present.

The following step (Fig. 1, fourth step) establishes the relation between a plant’s day and night nitrate uptake responses. The uptake flux of nitrate in several plant species is 3:2 between day and night (Maurice Cheung *et al.* 2014). Although these are the ratio values set as the default in the *diel_models’*s algorithm, they can also be changed or removed if desired. According to Forde (2002), this difference represents an adaptive strategy of plants to maximize resource consumption, by adapting nutrition intake based on light conditions and energy needs. Again, the flux of several reactions can change, as multiple nitrate uptake reactions may be present in a multi-tissue model.

Finally, optionally, a new reaction that merges the day and night biomass reactions may be created in the fifth step (Fig. 1, fifth step). Subsequently, the model objective function is set to the maximization of the total biomass reaction, and the flux boundaries of each individual biomass reaction are set to zero.

## 3 Validation methods and results

Several simulations were performed during the validation process of *diel_models* to evaluate its ability to represent plant metabolic changes across day–night cycles. Validation of the model was conducted for both generic and multi-tissue models, including AraGEM (de Oliveira Dal’Molin *et al.* 2010) for *A. thaliana* and a multi-tissue model of *Quercus suber* (Cunha *et al.* 2023), which comprises different tissues such as the leaf, inner bark, and phellogen. These simulations focused on verifying flux behaviours in diel versus non-diel models, particularly in light-dependent reactions. Additionally, reaction fluxes and Quantum Yield (QY) values across several non-diel and diel plant models, were analysed, comparing them to established literature values for verification. Also, to assess the impact of the empirical 3:2 nitrogen uptake ratio between day and night, we conducted additional simulations in which this constraint was not considered.

*diel_models* effectively replicated day and night metabolic fluxes for AraGEM, as indicated by photosynthesis and RuBisCO reaction fluxes (Supplementary Table S1) and Calvin Cycle reactions (Supplementary Table S2). Minimal night-phase activity was observed only in the ribulose diphosphate carboxylation and 1,3-BPG to G3P reactions. This confirms the diel model’s capability to accurately simulate plant-specific diel metabolic shifts.

In multi-tissue models, *diel_models* successfully ensured tissue-specific regulation of photon uptake and nitrate fluxes while preserving inter-tissue metabolic connectivity. The *Q. suber* model validated the preservation of tissue-specific diel behaviours, with near-zero fluxes in night-specific reactions (Supplementary Tables S3 and S4**)**, thereby accurately reflecting inter-tissue metabolic differences.

Exchange fluxes in storage pools were also simulated, considering that the storage pool metabolites are those described on Supplementary Data. This analysis reflects the expected metabolite cycling: compounds such as sucrose and starch flowed from day to night phases, while other metabolites like nitrate and alanine showed inverse flows, in both generic (AraGEM) and multi-tissue (*Q. suber*) models, aligning with documented plant metabolic cycles (Maurice Cheung *et al.* 2014). The direction and magnitude of these fluxes support *diel_models*’ reliable handling of diel metabolic transitions and can be found in more detail in Supplementary Tables S5 and S7.

The results of removing the 3:2 ratio showed that the primary effect of removing this empirical assumption was the elimination of nitrate flow from the night to the day phase (Supplementary Tables S6 and S8). This indicates that enforcing the ratio leads to an increased nitrate uptake during the night, necessitating its transport to the day phase. However, key model properties, such as quantum yield (Supplementary Table S10) and the fluxes of other reactions (Supplementary Tables S1–S4), remained largely unaffected, suggesting that this assumption primarily influences the temporal distribution of nitrate uptake rather than core metabolic processes. Given its limited impact on broader model behaviour, we retain this ratio as a default while ensuring that users can modify it based on their specific needs.

The AraGEM diel model was subsequently analysed using differential flux analysis (DFA), a routine previously used in Nanda and Ghosh (2021) and Sampaio *et al.* (2024), where the ACHR sampler (Bordel *et al.* 2010) was used to generate 100 sample fluxes for all reactions from the different phases. Then, these data were used to identify the reactions with differential fluxes between phases. Next, hypergeometric enrichment tests were used to identify the pathways with reactions presenting significantly differential flux between each phase, shown in Supplementary Fig. S2. The pathways with the highest number of statistically different reactions include fatty acid biosynthesis (52 out of 72), carbon fixation (24 out of 39), and glycolysis/gluconeogenesis (16 out of 17), in contrast to pathways such as steroid biosynthesis (3 out of 54) and flavonoid biosynthesis (1 out of 47). During daylight, chloroplasts harness light energy to synthesize ATP and NADPH, crucial for fatty acid biosynthesis (Rawsthorne 2002). Consequently, this pathway exhibits differential expression in flux between day and night, having more flux during the day. Glycolysis involves the conversion of glucose to pyruvate, producing ATP and NADH, which can occur continuously. In contrast, gluconeogenesis synthesizes glucose from metabolic precursors such as pyruvate, lactate, amino acids, or citric acid cycle intermediates, more actively at night when plants primarily engage in cellular respiration.

Principal Component Analysis (PCA), applied to the reactions that are differentially expressed, further illustrates distinct clustering of day and night flux patterns, emphasizing diel metabolic shifts (Supplementary Fig. S3). Together, these two components account for about 98% of the model’s variability. By setting a threshold of 1, defined as the maximum allowable Euclidean distance for two points to be considered overlapping, it was determined that only 15 out of the 523 (∼2.9%) reactions with statistically different fluxes overlapped between different phases (e.g. TCM21_Day and TCM21_Night), as shown in Supplementary Data, which it was considered to be a residual percentage. However, such an intersection is reasonable as certain metabolic activities remain unaffected by changes in light exposure. For instance, reactions involved in ascorbate and aldarate metabolism, as well as glyoxylate and dicarboxylate metabolism, were observed to overlap between day and night, indicating their independence from light-dependent regulation.

Supplementary Table S10 compares QY values for several non-diel and diel models (created by our tool) of different plant species (more detail on the models used and the modifications applied available in Supplementary Data). The QY represents the amount of CO_2_ fixed per mol of photons. For metabolic models, QY is calculated by dividing the flux of the RuBisCO carboxylation reaction by the flux of the photon uptake reaction, reflecting how efficiently the plant converts light energy into usable chemical energy. The QY reference values are sourced from the literature, while the estimated values are generated by the models. These results demonstrate that diel models generated by *diel_models* produced QY values more closely aligned with experimental references than their non-diel counterparts. Among the five models converted, only one did not match or come close to the literature values, while another exhibited QY within the reference range and, the remaining three, *Populus trichocarpa*, *Solanum lycopersicum* (2015), and *Solanum tuberosum*, showed values closer to actual measurements compared to their non-diel counterparts. These findings demonstrate the tool’s effectiveness in producing diel models that better simulate plant metabolism and photosynthetic efficiency.

While the proposed method effectively builds diel models, certain limitations should be acknowledged. Our current method remains agnostic to the duration of day and night, meaning that it does not yet account for variations in photoperiod due to seasonal or geographical differences. While this simplification allows for a more generalizable approach, the partitioning of metabolic fluxes—particularly those associated with maintenance energy demands—can be refined in future iterations. Specifically, if an ATP maintenance reaction is included, its flux should ideally be scaled according to the relative duration of each phase, assuming a constant maintenance cost per unit of time. Additionally, an uneven distribution of time between day and night would influence the absolute flux values, even if the specific rates (flux/time/mass) remain unchanged. This consideration can be incorporated as an additional step in the pipeline, allowing for a more precise representation of diel metabolic dynamics when necessary.

## 4 Conclusion

Integrating diel cycles into plant genome-scale metabolic models (pGEMs) poses challenges due to the lack of standardized and systematized pipelines within the community. In this study, we introduce *diel_models*, a novel fully automated method designed to transform non-diel pGEMs into diel pGEMs, with flexibility for community-driven refinements and expansions. This package has been tested for compatibility with Python versions 3.8 and above and works seamlessly across all operating systems. *diel_models* successfully incorporates day–night metabolism in both generic and multi-tissue pGEMs, approximating the estimated QY values with those reported in the literature. This advancement streamlines and accelerates the development of diel pGEMs, highlighting the importance of these models in offering more realistic and practical insights into organism metabolism.

## Supplementary Material

vbaf087_Supplementary_Data
