# Supplementary material for: diel_models: a python package for systematic integration of day–night cycles into plant genome-scale metabolic models
Source: Bioinform Adv. 2025 Apr 16;5(1):vbaf087. doi: 10.1093/bioadv/vbaf087 (PMC12070391; doi:10.1093/bioadv/vbaf087)
Supplement: vbaf087_Supplementary_Data [file vbaf087_supplementary_data.zip › Appendix_DielModels_final.pdf]

Supplementary Material - *diel_models*

Table 1: Details about the fluxes (mmol/hour/[gram dry weight]) in the diel model in the day and night phases, as well as the original model - AraGEM's photosynthesis, carboxylation, and oxygenation of RuBisCO reactions.

Model	Phase	Photosynthesis Reaction	Ribulose Biphosphate Carboxylation	Ribulose Biphosphate Oxygenation
**Diel**	*Day*	8.149	7.149	2.383
	Night	0.000	0.023	0.000
**Diel (no nitrate uptake restriction)**	*Day*	8.149	7.149	2.383
	Night	0.000	0.000	0.000
**Non-Diel**	-	8.149	7.149	2.383

Table 2: Details on the day-night fluxes (mmol/hour/[gram dry weight]) in the AraGEM diel model and the original model for the Calvin Cycle reactions. 3-PGA (3-Phosphoglyceric acid), 1,3-BPG (1,3-Bisphosphoglyceric acid), G3P (Glyceraldehyde 3-phosphate), R5P (Ribose 5-phosphate), Ru5P (Ribulose 5-phosphate), RuBP (Ribulose 1,5-bisphosphate).

Model	Phase	3-PGA to 1,3-BPG	1,3-BPG to G3P	G3P to R5P	R5P to Ru5P	Ru5P to RBP
**Diel**	*Day*	14.192	-13.897	3.177	3.177	9.532
	Night	0.000	0.001	0.000	0.000	0.000
**Diel (no nitrate uptake restriction)**	*Day*	13.924	-13.629	3.177	3.177	9.532
	Night	0.000	0.000	0.000	0.000	0.000
**Non-Diel**	-	14.185	-13.620	3.177	3.177	9.532

Table 3: Details about the fluxes (mmol/hour/[gram dry weight]) in the diel multi-tissue model between day and night, as well as the original multi-tissue model - *Q. suber*'s photosystem I, carboxylation, and oxygenation of RuBisCO reactions.

Model	Phase	Photosystem I	Ribulose Biphosphate Carboxylation	Ribulose Biphosphate Oxygenation
**Diel**	*Day*	48.206	7.863	2.621
	Night	0.000	0.008	0.003
**Diel (no nitrate uptake restriction)**	*Day*	48.206	7.863	2.621
	Night	0.000	0.008	0.003
**Non-Diel**	-	2.351	0.327	0.109

Table 4: Details on the day-night fluxes (mmol/hour/[gram dry weight]) in the *Q. suber* multi-tissue diel model and the original model for the Calvin Cycle reactions. 3-PGA (3-Phosphoglyceric acid), 1,3-BPG (1,3-Bisphosphoglyceric acid), G3P (Glyceraldehyde 3-phosphate), GP (Glycerone phosphate), SBP (Sedoheptulose1,7-bisphosphate), S7P (Sedoheptulose7-phosphate), R5P (Ribose 5-phosphate), Ru5P (Ribulose 5-phosphate), RuBP (Ribulose 1,5-bisphosphate)

**Model**	**Phase**	**3-PGA to 1,3-BPG**	**1,3-BPG to G3P**	**G3P to GP**	**GP to SBP**	**SBP to S7P**	**S7P to R5P**	**R5P to Ru5P**	**Ru5P to RBP**
**Diel**	*Day*	21.164	-21.164	7.511	3.470	3.470	3.470	3.470	10.484
	Night	-0.264	0.264	-0.274	0.000	0.000	0.000	-0.001	0.011
**Diel (no nitrate uptake restriction)**	*Day*	21.164	-21.164	7.511	3.470	3.470	3.470	3.470	10.484
	Night	-0.264	0.264	-0.274	0.000	0.000	0.000	-0.001	0.011
**Non-Diel**	-	1.043	-1.043	0.304	0.145	0.145	0.145	0.145	0.436

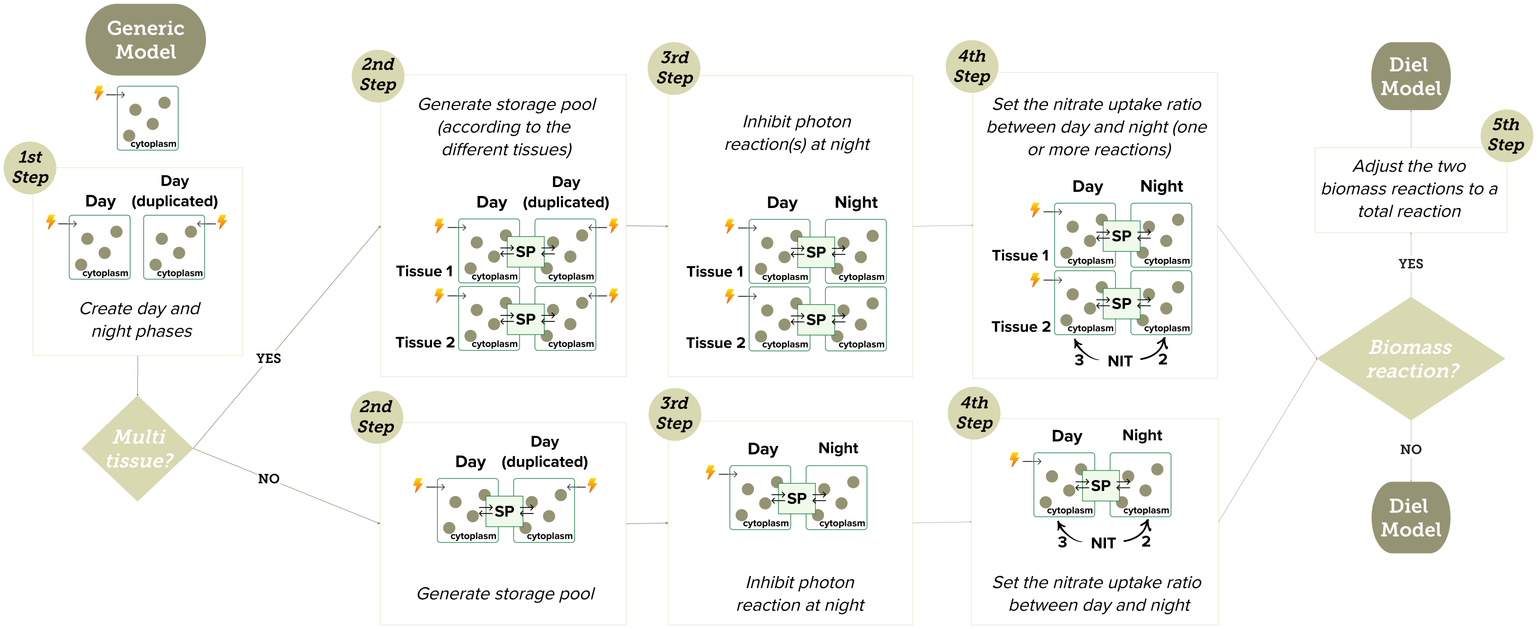

Figure 1: Diel models creator detailed pipeline illustration.

Table 5: Information about the flow of metabolite exchange reactions between (the storage pool) and day-night phases in the diel AraGEM model.

**Positive pFBA Flux (Day → Night)**	**Negative pFBA Flux (Night → Day)**
Sucrose	Nitrate
Starch	Citrate
Malate	Fructose
Fumarate	Alanine
Isoleucine	Glycine
Leucine	
Lysine	
Methionine	
Phenylalanine	
Threonine	
Tryptophan	
Valine	
Cysteine	
Glutamine	
Glutamate	
Tyrosine	
Asparagine	
Serine	
Aspartate	

A positive flux indicates that the compound moves from the day phase to the storage pool and, ultimately, to the night phase. On the other hand, those with negative flux go from the night phase to the storage pool to transition to the day phase.

References

[1] Kelly Botero, Silvia Restrepo, and Andres Pinzón. "A genome-scale metabolic model of potato late blight suggests a photosynthesis suppression mechanism". In: *BMC Genomics* 19.8 (2018).

[2] James Ehleringer and Robert W Pearcy. "Variation in Quantum Yield for CO2 Uptake among C3 and C4 Plants'". In: *Plant Physiol* 73 (1983), pp. 555–559.

[3] Léo Gerlin et al. "A multi-organ metabolic model of tomato predicts plant responses to nutritional and genetic perturbations". In: *Plant Physiology* 188.3 (Nov. 2021), pp. 1709–1723.

[4] J. Aaron Hogan et al. "The physiological acclimation and growth response of Populus trichocarpa to warming". In: *Physiologia Plantarum* 173 (3 Nov. 2021), pp. 1008–1029.

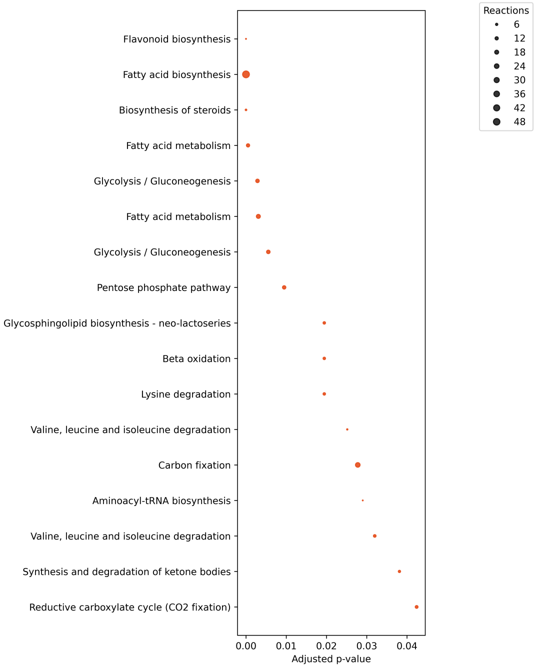

Figure 2: Plot from the pathway enrichment method representing the number of differential reactions between day and night in each pathway - AraGEM.

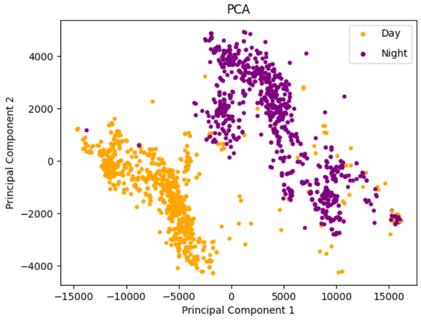

Figure 3: PCA plot, with the sampling values filtered by the differentially expressed reactions - AraGEM.

Table 6: Information about the flow of metabolite exchange reactions between (the storage pool) and day-night phases in the diel AraGEM model without applying the nitrate exchange reaction constraint.

**Positive pFBA Flux (Day → Night)**	**Negative pFBA Flux (Night → Day)**
Sucrose	Citrate
Starch	Fructose
Malate	Alanine
Fumarate	Glycine
Isoleucine	
Leucine	
Lysine	
Methionine	
Phenylalanine	
Threonine	
Tryptophan	
Valine	
Cysteine	
Glutamine	
Glutamate	
Tyrosine	
Asparagine	
Serine	
Aspartate	

[5] Russell K Monson, Robert O Littlejohn, and George J Williams. *The quantum yield for C02 uptake in Ca and C4 grasses*. 1982.

[6] R Saha, P F Suthers, and C D Maranas. “Zea mays iRS1563: A Comprehensive Genome-Scale Metabolic Reconstruction of Maize Metabolism”. In: *PLoS ONE* 6.7 (2011), p. 21784.

[7] Debolina Sarkar and Costas D. Maranas. “SNPeffect: identifying functional roles of SNPs using metabolic networks”. In: *Plant Journal* 103.2 (2020), pp. 512–531.

[8] Xinyou Yin et al. “Accounting for the decrease of photosystem photochemical efficiency with increasing irradiance to estimate quantum yield of leaf photosynthesis”. In: *Photosynthesis Research* 122 (2014), pp. 323–335.

[9] Huili Yuan et al. “A genome-scale metabolic network reconstruction of tomato (Solanum lycopersicum L.) and its application to photorespiratory metabolism”. In: *Plant Journal* 85.2 (2016), pp. 289–304.

Table 7: Information about the flow of metabolite exchange reactions between (the storage pool) and day-night phases in the multi-tissue diel *Quercus suber* model. Ibark - Inner Bark.

**Positive pFBA Flux (Day → Night)**	**Negative pFBA Flux (Night → Day)**
Leaf and Phellogen Sulfate	Leaf and Phellogen Sucrose
Leaf Nitrate	Ibark Nitrate
Leaf, Ibark and Phellogen Histidine	Leaf Leucine
Leaf, Ibark and Phellogen Isoleucine	Leaf Threonine
Leaf, Ibark and Phellogen Methionine	Leaf Valine
Leaf, Ibark and Phellogen Arginine	Leaf and Ibark Glutamine
Leaf Glycine	Ibark and Phellogen Glycine
Leaf, Ibark and Phellogen Proline	Leaf and Phellogen Glutamate
Leaf, Ibark and Phellogen Tyrosine	Leaf and Ibark Asparagine
Ibark Glutamate	Leaf Serine
Phellogen Serine	Leaf Fumarate
Ibark and Phellogen Fructose	
Ibark and Phellogen Fumarate	
Ibark and Phellogen Citrate	
Leaf and Ibark Starch	

Table 8: Information about the flow of metabolite exchange reactions between (the storage pool) and day-night phases in the multi-tissue diel *Quercus suber* model without applying the nitrate exchange reaction constraint.. Ibark - Inner Bark.

**Positive pFBA Flux (Day → Night)**	**Negative pFBA Flux (Night → Day)**
Leaf and Phellogen Sulfate	Leaf and Phellogen Sucrose
Leaf Nitrate	Leaf Fumarate
Leaf, Ibark and Phellogen Histidine	Leaf Leucine
Leaf, Ibark and Phellogen Isoleucine	Leaf Threonine
Leaf, Ibark and Phellogen Methionine	Leaf Valine
Leaf, Ibark and Phellogen Arginine	Leaf and Ibark Glutamine
Leaf Glycine	Ibark and Phellogen Glycine
Leaf, Ibark and Phellogen Proline	Leaf and Phellogen Glutamate
Leaf, Ibark and Phellogen Tyrosine	Leaf and Ibark Asparagine
Ibark Glutamate	Leaf Serine
Phellogen Serine	
Ibark and Phellogen Fructose	
Ibark and Phellogen Fumarate	
Ibark and Phellogen Citrate	
Leaf and Ibark Starch	

Table 9: Information about the flow of metabolite exchange reactions between (the storage pool) and day-night phases in the multi-tissue diel *Quercus suber* model without nitrate uptake constraint. Ibark - Inner Bark.

**Positive pFBA Flux (Day → Night)**	**Negative pFBA Flux (Night → Day)**
Leaf and Phellogen Sulfate	Leaf and Phellogen Sucrose
Leaf Nitrate	Leaf Citrate
Leaf, Ibark and Phellogen Histidine	Leaf Leucine
Leaf, Ibark and Phellogen Isoleucine	Leaf Threonine
Leaf, Ibark and Phellogen Methionine	Leaf Valine
Leaf, Ibark and Phellogen Arginine	Leaf and Ibark Glutamine
Leaf Glycine	Ibark and Phellogen Glycine
Leaf, Ibark and Phellogen Proline	Leaf and Phellogen Glutamate
Leaf, Ibark and Phellogen Tyrosine	Leaf and Ibark Asparagine
Ibark Glutamate	Leaf Serine
Phellogen Serine	Leaf Fumarate
Ibark and Phellogen Fructose	
Ibark and Phellogen Fumarate	
Ibark and Phellogen Citrate	
Leaf and Ibark Starch	

Table 10: Comparison between the quantum yield values for several non-diel and diel models.

**Model**	**Quantum Yield**	
	estimate	*reference*
P. trichocarpa [7] non-diel	0.100	0.015 - 0.025 [4]
Diel *P. trichocarpa* (ours)	0.063	
Diel *P. trichocarpa* (ours w/o nitrate uptake restriction)	0.063	
S. lycopersicum 2015 [9] non-diel	0.131	0.027-0.072 [8]
Diel *S. lycopersicum* 2015 (ours)	0.106	
Diel *S. lycopersicum* 2015 (ours w/o nitrate uptake restriction)	0.106	
S. lycopersicum 2022 [3] non-diel	0.002	
Diel *S. lycopersicum* 2022 (ours)	0.00006	
Diel *S. lycopersicum* 2022 (ours w/o nitrate uptake restriction)	0.002	
Z. mays [6] non-diel	0.033	0.050 - 0.062 [2]
Diel *Z. mays* (ours)	0.059	
Diel *Z. mays* (ours w/o nitrate uptake restriction)	0.059	
S. tuberosum [1] non-diel	0.210	0.045-0.068 [5]
Diel *S. tuberosum* (ours)	0.119	
Diel *S. tuberosum* (ours w/o nitrate uptake restriction)	0.119	
